# Lactate inhibits glucose‐induced zigzag motility and enhances linear motility in bull spermatozoa by suppressing glycolysis

**DOI:** 10.1111/andr.70113

**Published:** 2025-08-15

**Authors:** Md Faizul Hossain Miraz, Takahiro Yamanaka, Shahrina Akter, Masanori Koyago, Masayuki Shimada

**Affiliations:** ^1^ Graduate School of Integrated Sciences for Life Hiroshima University Higashihiroshima Hiroshima Japan; ^2^ Bangladesh Livestock Research Institute Savar, Dhaka Bangladesh; ^3^ Livestock Improvement Association of Japan Inc Tokyo Japan; ^4^ Graduate School of Innovation and Practice for Smart Society Hiroshima University Higashihiroshima Hiroshima Japan

**Keywords:** acrosome reaction, glucose, glycolysis, lactate, motility, spermatozoa

## Abstract

**Background:**

Energy metabolism and substrate balance are critical determinants of sperm motility and fertility. Linear motility is necessary for sperm forward movement, whereas hyperactivated motility is a prerequisite for fertilization. The preference of metabolic pathways depends on substrate availability which controls sperm motility. However, there are differences in substrate composition in seminal plasma, vagina, uterus and oviducts.

**Objectives:**

This study aims to clarify how the spermatozoa maintains its metabolic homeostasis and functions in the presence of glucose and lactate either alone or in combinations.

**Materials and Methods:**

Fresh bull spermatozoa was incubated with modified human tubal fluid (mHTF) medium containing either no‐energy, glucose, lactate, or a combination of glucose and lactate. Sperm motility, kinematics, adenosine triphosphate (ATP) production, mitochondrial membrane potential, glucose incorporation, glycolysis, oxidative phosphorylation (OXPHOS), and acrosome reaction were systematically assessed.

**Results:**

Glucose resulted in zigzag motility and lactate induced linear motility. Glucose‐derived zigzag motility was suppressed by lactate with increasing lactate concentration in a dose‐dependent manner. The addition of lactate with glucose showed higher mitochondrial membrane potential, higher oxygen consumption rate (OCR), and lower extracellular acidification rate (ECAR). Lactate suppressed glucose incorporation in midpiece and tail regions, reduced glycolysis, and shifted sperm metabolism toward OXPHOS which resulted in linear motility and maintained acrosome integrity.

**Discussion:**

Lactate played a metabolic and regulatory role in bull sperm metabolism. As a metabolic role, it oxidized through OXPHOS and maintained linear motility. The metabolic changes by lactate suppressed glucose‐induced acrosome reactions and maintained linear motility, which might be beneficial for sperm transportation toward the fertilization site of the female reproductive tract and results in successful fertilization.

**Conclusion:**

This is a novel finding that explores the regulatory role of lactate over glucose metabolism in bull sperm functionality. Optimum balancing of glucose and lactate maintains the motility and functionality of bull spermatozoa. These outcomes might have substantial implications for the enhancement of sperm preservation techniques, sperm handling, and fertility outcomes.

## 1 INTRODUCTION

Mammalian spermatozoa are produced in the testes and stored in the epididymis in a quiescent state.^1^ After ejaculation, spermatozoa mix with seminal plasma, which activates motility and transports the spermatozoa through the uterus and oviducts to reach the fertilization site.^2^ During this transit, spermatozoa navigate various dynamic microenvironments,^3^ including seminal plasma, vaginal fluid, uterine fluid, and oviductal fluid. However, ion concentrations and metabolite compositions are different in each of these segments.^4^, ^5^ Differences in metabolic substrates and oxygen concentration drive the spermatozoa to balance between glycolysis and oxidative phosphorylation (OXPHOS) to generate sufficient adenosine triphosphate (ATP) for motility, capacitation and fertilization.^6^, ^7^

The metabolic preferences of spermatozoa differ among the species.^8^ Glycolysis is the predominant metabolic pathway in humans^9^ and rodent spermatozoa,^10^ while OXPHOS serves as the prime metabolic pathway in horses.^11^ In bull spermatozoa, ATP synthesis is facilitated by an interplay of both glycolysis and OXPHOS,^12^ making their energy homeostasis more complex. Bull spermatozoa can use both types of substrates to produce ATP.^13^ The acquisition of this ability means that bull spermatozoa already retain enzymes involved in glycolysis and OXPHOS in mitochondria during the spermatogenesis process, and can use them to produce ATP.^14^, ^15^ However, despite having this ability, it does not motile in the epididymis during the sperm maturation process.^1^ In addition, although motility is exerted by ejaculation, the spermatozoa are exposed to different energy conditions in semen, vagina, cervix, uterus, and fallopian tubes to reach the site of fertilization.^16^ It has been reported that ATP produced at different sites changes the motility pattern of spermatozoa.^2^ In addition, because the pattern of motility changes depending on the amount of ATP produced, this difference in the composition of this complex nutrient substrate may have a significant impact on sperm motility patterns, but the details of this point are unclear.

In this study, we investigated how spermatozoa exhibits different motility patterns when exposed to different energy substrates alone or in combination. The spermatozoa was treated with glucose, lactate, and a combination of glucose and lactate to observe the motility, kinematics and functionality. Our findings provide a novel insight into the lactate's dual role in sperm metabolism. We propose that lactate functions as an effective energy substrate while simultaneously modulating glucose metabolism, improving linear motility, and inhibiting early acrosome reaction. Lactate's capacity to inhibit glucose‐induced acrosome reactivity may function as a control mechanism to prevent early acrosome reactions before spermatozoa encounters the oocyte. Comprehending these metabolic connections could greatly influence the enhancement of sperm handling and preservation methods and eventually improve fertility outcomes.

## 2 MATERIALS AND METHODS

### 2.1 Chemicals and reagents

All chemicals and reagents used in this experiment were purchased from Sigma‐Aldrich, Nacalai Tesque, or FUJIFILM Wako Pure Chemical Corporation.

### 2.2 Animals and semen collection

Fresh semen was collected from three healthy and mature Japanese black bulls provided by the Livestock Improvement Association of Japan, INC. The experiment was conducted with proper approval from the Animal Care and Use Committee of Hiroshima University (Approval number: E 23‐4). Semen was collected by the artificial vagina method and diluted with Tris–egg yolk citrate extender. The diluted semen was maintained at 4°C and transported to the laboratory within 4 h for analysis.

### 2.3 Sperm preparation and incubation

**FIGURE 1:**
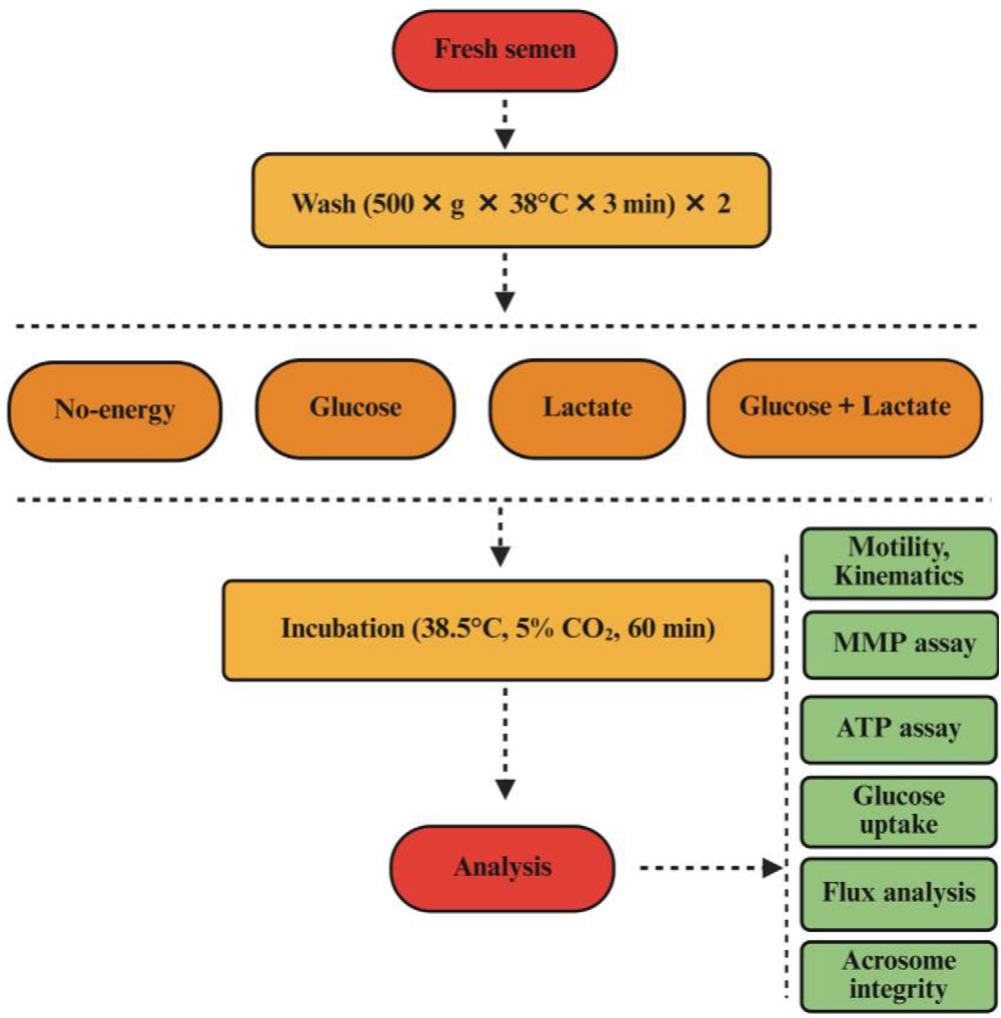
Schematic presentation of experimental design. Fresh bovine semen was washed with no‐energy modified human tubal fluid (mHTF) medium and incubated for 60 min with different substrates (no‐energy, glucose, lactate, and glucose + lactate). Sperm motility, kinematics, adenosine triphosphate (ATP) assay, mitochondrial membrane potential (MMP), glucose uptake, flux analysis, and acrosome integrity were assessed to understand the role of substrates on sperm motility and functionality.

Fresh semen was washed with modified human tubal fluid (mHTF).^17^ mHTF was prepared with either no‐energy, glucose, Na‐lactate, or a combination of glucose and Na‐lactate and termed as “no‐energy,” “glucose,” “lactate,” and “glucose + lactate.” Glucose and Na‐lactate concentration of the medium was 2.8 mM and 23.6 mM, respectively (Table S1). The glucose (2.8 mM) and lactate (23.6 mM) concentrations in our experiment were determined based on the commercially available composition of human tubal fluid (HTF) medium. These values overlap with the range of bovine oviduct fluid (2–6 mM glucose and 5–10 mM lactate) and the widely used Biggers‐Whitten‐Whittingham (BWW) fertilization‐enhancing medium (5.56 mM glucose and 21.6 mM lactate). In the dose‐dependent study, a Na‐lactate concentration of 23.6 mM, 2.36 mM, and 0.236 mM was considered as 100%, 10%, and 1% Na‐lactate, respectively. The osmolarity was maintained at 300 ± 5 mOsm, and pH was adjusted at 7.4 across the treatments. Additional NaCl was used to modify osmolarity as required. A minimum total motility of 60% was considered as standard for the experiment. About 20–30 µL of fresh semen was mixed with 1000 µL of no‐energy medium to adjust about 20 million/mL sperm concentration. The sample was washed two times at 500 × *g* for 3 min at 38°C with no‐energy medium. The washed semen was subsequently incubated in a humidified incubator at 38.5°C with 5% CO_2_ for 1 h in no‐energy, glucose, lactate, and glucose + lactate medium. The detailed experimental layout is presented in Figure 1.

### 2.4 Sperm motility and kinematics assay

Sperm motility and kinematics were evaluated by a computer‐aided sperm analysis (CASA).^18^ In brief, 3 µL of diluted semen sample was used for observing by pre‐warmed counting Leja slides. Sperm tracks (0.5 s, 45 frames) were recorded at 60 Hz with the CASA system (HT CASA‐Ceros II; Hamilton Throne). Motile spermatozoa, denoted by an average path velocity of >4 µm/s and a straight‐line velocity of >1 µm/s were considered for motility and individual sperm kinematics assessment. Straight‐line velocity (VSL), curvilinear velocity (VCL), amplitude of lateral head displacement (ALH), and linearity (LIN) were considered for sperm kinematics. In this experiment, higher VCL and ALH were considered as an indication of zigzag motility whereas, higher VSL and lower VCL and ALH were considered as linear motility. More than 200 trajectories were recorded in a minimum of five fields.

### 2.5 ATP localization in spermatozoa

Fresh spermatozoa were incubated with different energy substrates (no‐energy, glucose, lactate, and glucose + lactate) and ATP localization was measured with BioTracker ATP‐Red (SCT045, Sigma‐Aldrich). In brief, the spermatozoa was washed two times with no‐energy medium. After washing, the spermatozoa was incubated for 15 min with 1000 µL of glucose, lactate and glucose + lactate medium. The spermatozoa was incubated in the dark for an additional 15 min with 2 µL of BioTracker ATP‐Red and washed once after incubation. The sample was resuspended with no‐energy medium and the percentage of ATP‐red positive spermatozoa and mean fluorescence intensity were analyzed using flow cytometry (Attune™ CytPix, Thermo Fisher Scientific Inc.) with a 488 nm laser and a filter with a bandwidth of 574/26 nm. ATP localization was evaluated by a confocal microscope (Nikon AX, Nikon Corporation). The gating technique for flow cytometry is presented in Figure S1.

### 2.6 Evaluation of mitochondrial membrane potential

Mitochondrial membrane potential (MMP) was evaluated with MitoPT® JC‐1 Assay Kit (Immuno Chemistry Technologies, LLC) in accordance to our previous study.^19^ Fresh semen was washed with no‐energy medium and subsequently incubated with 200 µL of glucose, lactate, and glucose + lactate medium for 15 min. After incubation, 2 µL of JC‐1 dye was added to each treatment group, and the samples were incubated for an additional 15 min in the dark. The sample was reconstituted with the respective medium after washing and MMP was assessed by flow cytometry with a 488 nm laser and a filter with a bandwidth of 574/26 nm.

### 2.7 Glucose uptake assay

Glucose uptake was detected by Glucose Uptake Assay Kit‐Green (UP02, Dojindo Laboratory). In brief, fresh semen was washed with no‐energy medium and incubated for 30 min with 1000 µL of no‐energy and lactate medium. Pre‐warmed probe working solution (2 µL) was added to the medium and incubated for another 15 min by wrapping it with aluminum foil. After incubation, 300 µL of 0.4% Trypan blue solution was added for quenching and further incubated for 5 min. The supernatant was removed by centrifugation and washed with Washing and Imaging Solution (WI solution) until the color changed. Finally, WI solution was added and observed by flow cytometry with a 488 nm laser and 530/30 nm bandwidth. Fluorescein Isothiocyanate (FITC)‐positive spermatozoa that had taken up fluorescent‐labeled glucose were observed under the confocal microscope. The observed spermatozoa were classified into four positive staining patterns: head, midpiece (MP), head + MP, and head + MP + tail. The positive percentage for each pattern was calculated by dividing the number of spermatozoa in that pattern by the total number of spermatozoa.

### 2.8 Extracellular flux analysis

The oxygen consumption rate (OCR) and extracellular acidification rate (ECAR) were determined using an extracellular flux analyzer (XF HS Mini; Agilent Technologies). The culture plate of the flux analyzer was coated with 20 µL of Concanavalin A (0.5 mg/mL, Fujifilm Wako Chemicals). Dilution buffer was added to each well of the culture plate and incubated overnight in a CO_2_^−^ free incubator. NaHCO_3_^−^ free no‐energy medium (1 mM HEPES) was used to wash bovine spermatozoa. Concanavalin A‐treated plates were seeded with 30 million sperm cells containing 180 µL of glucose, lactate, and glucose + lactate medium. The plates were centrifuged twice at 900 × *g* for 1 min, incubated at 37°C in 100% air, and analyzed for 1 h. Agilent Seahorse Wave software was used to quantify basal OCR and ECAR.

### 2.9 Acrosome integrity evaluation

Acrosome integrity of fresh spermatozoa and spermatozoa incubated with substrate was evaluated with a combination of probe lectin from *Arachis hypogea* (peanut) agglutinin attached to fluorescein (PNA‐FITC; L7381, Sigma‐Aldrich) and propidium iodide (PI; L 7011, Invitrogen). For this purpose, 0.025 mg/mL of PNA‐FITC and 2.4 nM of PI were mixed with 200 µL of no‐energy, glucose, lactate and glucose + lactate medium and incubated for 10 min at 38.5°C in a humidified incubator in the dark. The fluorescence was assessed by flow cytometry of 488 nm lesser with a bandwidth of 530/30 for PNA‐FITC and 695/40 for PI.

### 2.10 Statistical analysis

Data normality and homogeneity of variance were tested by Shapiro–Wilk test and Bartlett's test before conducting statistical analysis. GraphPad Prism (version 8.0.2) was used to conduct statistical analyses. The scatter plot was prepared using R (version 4.3.0). Each experiment was conducted with a minimum of three animal replicates. An unpaired Student's *T*‐test was conducted to compare the control and treatment groups. Differences among the treatment groups were evaluated by one‐way anova followed by Tukey HSD post hoc test. Data other than box plots were presented as mean ± SD. Statistical significance was considered when *p* < 0.05.

## 3 RESULTS

### 3.1 Glucose induces zigzag motility and lactate results in linear motility

**FIGURE 2:**
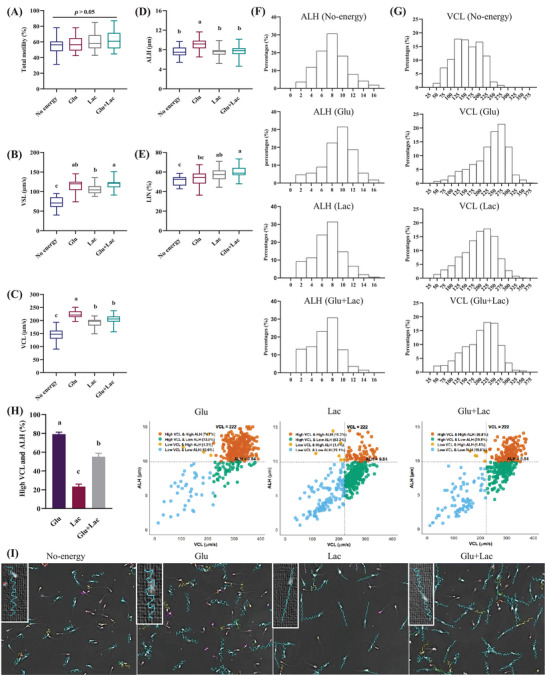
Glucose induces zigzag motility and lactate results in linear motility. Fresh spermatozoa was treated with no‐energy, glucose, lactate, and glucose + lactate for 60 min. Sperm motility and kinematics was evaluated with CASA. Box plots show total motility (%), VSL (µm/s), VCL (µm/s), ALH (µm), and LIN (%) of spermatozoa incubated with no‐energy, glucose, lactate, and glucose + lactate (A–E). Histogram peak shifts from right to left of ALH (µm) and VCL (µm/s) when lactate was added with glucose (F, G). Scatter diagram shows reduction of high VCL and ALH spermatozoa (%) when lactate was added with glucose (H). Variations in motility tracks with different substrate treatment observed with CASA (I). Data are presented from at least three replicates. Different lowercase subscripts indicate significant differences (*p* < 0.05). ALH, amplitude of lateral head displacement; CASA, computer‐aided sperm analysis; Glu, glucose; Lac, lactate; VCL, curvilinear velocity; VSL, straight line velocity.

The motility assay was performed by CASA to understand whether the spermatozoa showed different motility and kinematics when treated with no‐energy, glucose, lactate, and glucose + lactate, respectively. There were no significant differences in sperm motility after 1‐h incubation, irrespective of the substrate (Figure 2A); however, their kinematics differed significantly (*p* < 0.05). Zigzag pattern‐like motility as characteristics with higher VCL, ALH, and lower LIN was observed when glucose was used as a substrate. Lactate showed a linear motility pattern with lower VCL, ALH and higher VSL and LIN. Interestingly, the combinations of substrate showed reduced VCL and ALH (*p* < 0.05) than the glucose alone (Figure 2B–E). For a clear understanding, we have analyzed individual sperm kinematics irrespective of treatment. From the histogram data, both the VCL and ALH peaks were shifted from right to left when we combined lactate with glucose as compared with the glucose alone (Figure 2F,G). A scatter diagram was prepared considering VCL and ALH of the no‐energy medium as a threshold (VCL = 174.05 and ALH = 6.27). Scatter diagrams also align with histogram data, where spermatozoa with higher VCL and ALH were significantly reduced with the addition of lactate than glucose alone (Figure 2H,I).

### 3.2 Lactate suppresses glucose‐induced zigzag motility in a dose‐dependent manner

**FIGURE 3:**
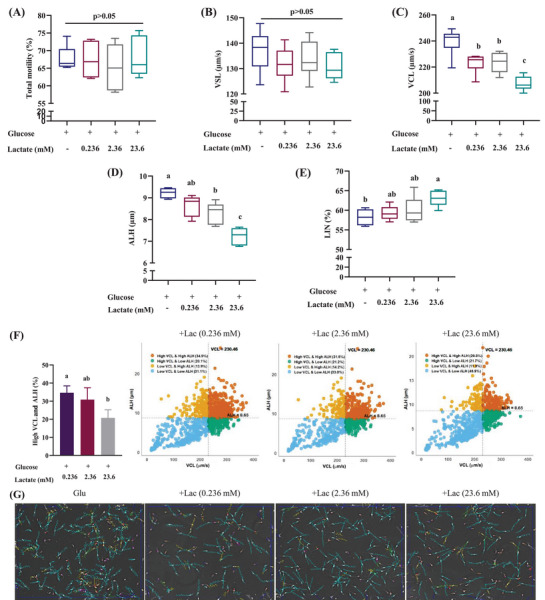
Lactate suppresses glucose‐induced zigzag motility in a dose‐dependent manner. Lactate was added with glucose at 1% (0.236 mM), 10% (2.36 mM), and 100% (23.6 mM) dose. Box plots show total motility (%), VSL (µm/s), VCL (µm/s), ALH (µm) and LIN (%) of spermatozoa incubated with glucose, glucose + 1% lactate, glucose + 10% lactate, and glucose + 100% lactate (A–E). Scatter diagram shows reduction of high VCL and ALH spermatozoa (%) at dose‐dependent manner with lactate addition (F). Variations in motility tracks with different doses of lactate observed with CASA (G). Data are presented from at least three replicates. Different lowercase subscripts indicate significant differences (*p* < 0.05). ALH, amplitude of lateral head displacement; CASA, computer‐aided sperm analysis; Glu, glucose; Lac, lactate; VCL, curvilinear velocity; VSL, straight line velocity.

Motility assay showed that lactate suppressed glucose‐induced zigzag motility (e.g., higher VCL and ALH; Figure 3A–E). So, we were curious to observe whether this suppression was dependent on the concentration of lactate. For a dose‐dependent study, 0.236, 2.36, and 23.6 mM lactate were considered as 1%, 10%, and 100% lactate, respectively. Lactate was found to suppress the glucose‐induced zigzag motility in a dose‐dependent manner (*p* < 0.05), as higher lactate doses result in lower VCL and ALH than the glucose alone (Figure 3C–E). A scatter diagram was prepared considering VCL and ALH of the glucose medium as a threshold (VCL = 230.46, ALH = 8.65). The scatter diagram showed that higher VCL and ALH percentages were reduced significantly with the increasing doses of lactate (Figure 3F,G).

### 3.3 Lactate addition with glucose results in higher OXPHOS, which yield elevated ATP and increased MMP

**FIGURE 4:**
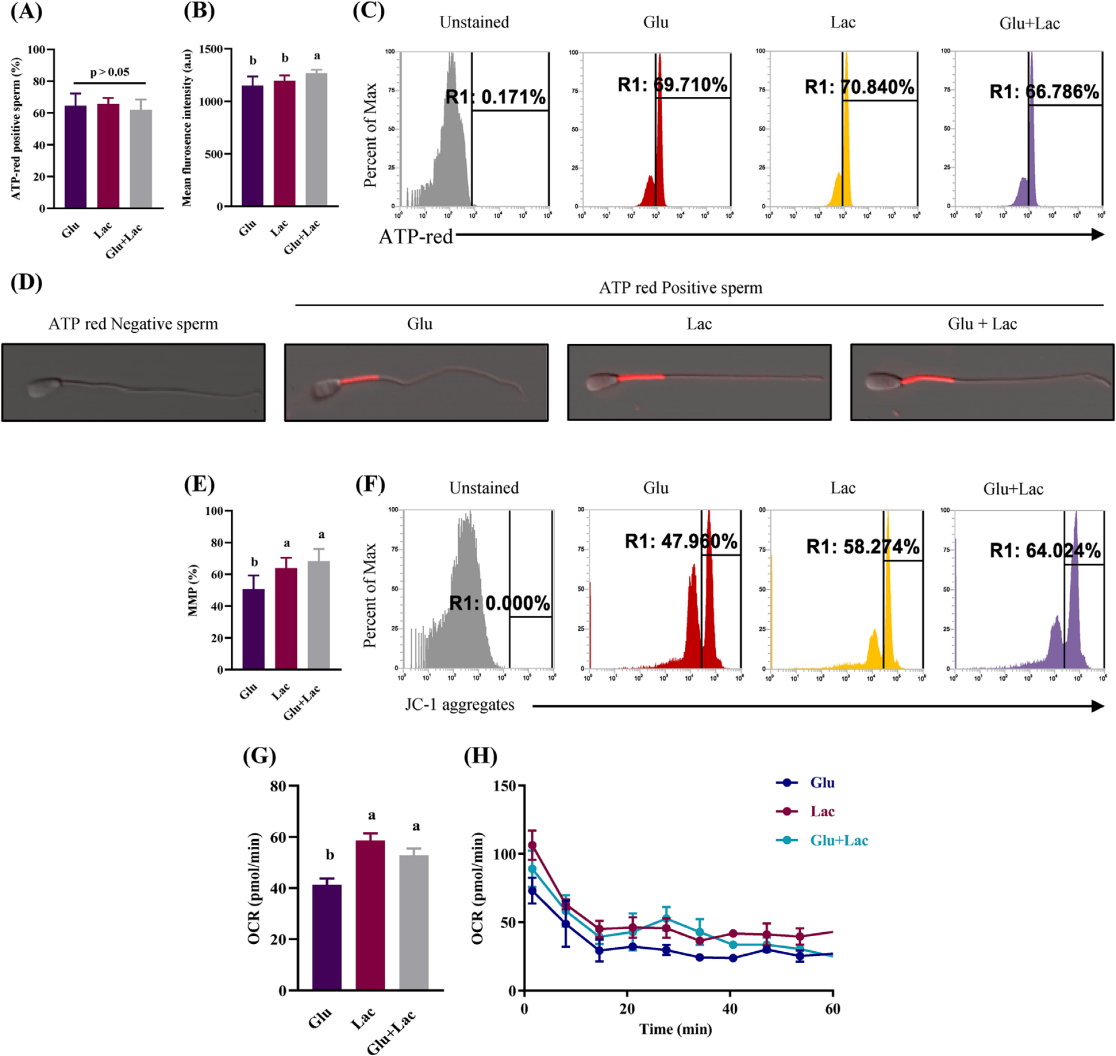
Lactate addition with glucose results in higher oxidative phosphorylation (OXPHOS), which yield elevated adenosine triphosphate (ATP) and increased MMP. Fresh bovine spermatozoa was incubated with glucose, lactate, and glucose + lactate and ATP‐red positive spermatozoa (%) and mean fluorescence intensity (a.u.) was assessed by flow cytometry (A–C). ATP localization was observed with confocal microscopy (D). Mitochondrial membrane potential (%) and mitochondrial OCR (pmol/min) were evaluated by JC‐1 staining (E, F) and flux analysis (G, H). Data are represented as mean ± SD from three replicates. Different lowercase subscripts indicate significant differences (*p* < 0.05). ATP, adenosine triphosphate; a.u., arbitrary units; Glu, glucose; Lac, lactate; MMP, mitochondrial membrane potential; OCR, oxygen consumption rate.

Sperm kinematics changes with the differences in substrate. To understand the role of substrates in sperm ATP production, we evaluated the ATP‐positive sperm percentages under different substrate conditions. Flow cytometry data showed that percentages of spermatozoa positive for ATP‐Red did not differ when spermatozoa were treated with alternative substrates; however, fluorescence intensity was higher when glucose was combined with lactate as compared with glucose or lactate alone (*p* < 0.05; Figure 4A–C). ATP was localized in the midpiece region of the spermatozoa (Figure 4D). These data indicated that the ability to produce ATP was not affected by the external substrate. However, the “ATP‐positive” gate had binary information. When the level of ATP red fluorescence in the mitochondria exceeded a low threshold, additional ATP only increased the intensity; it did not affect the number of positive cells. Reflecting ATP production in the midpiece, addition of lactate with glucose maintained the higher MMP (*p* < 0.05; Figure 4E,F). Flux analysis showed higher OCR in glucose + lactate than glucose alone (*p* < 0.05), indicating higher OXPHOS when both substrates were present in the medium (Figure 4G,H).

### 3.4 Lactate suppresses glucose uptake and glycolysis

**FIGURE 5:**
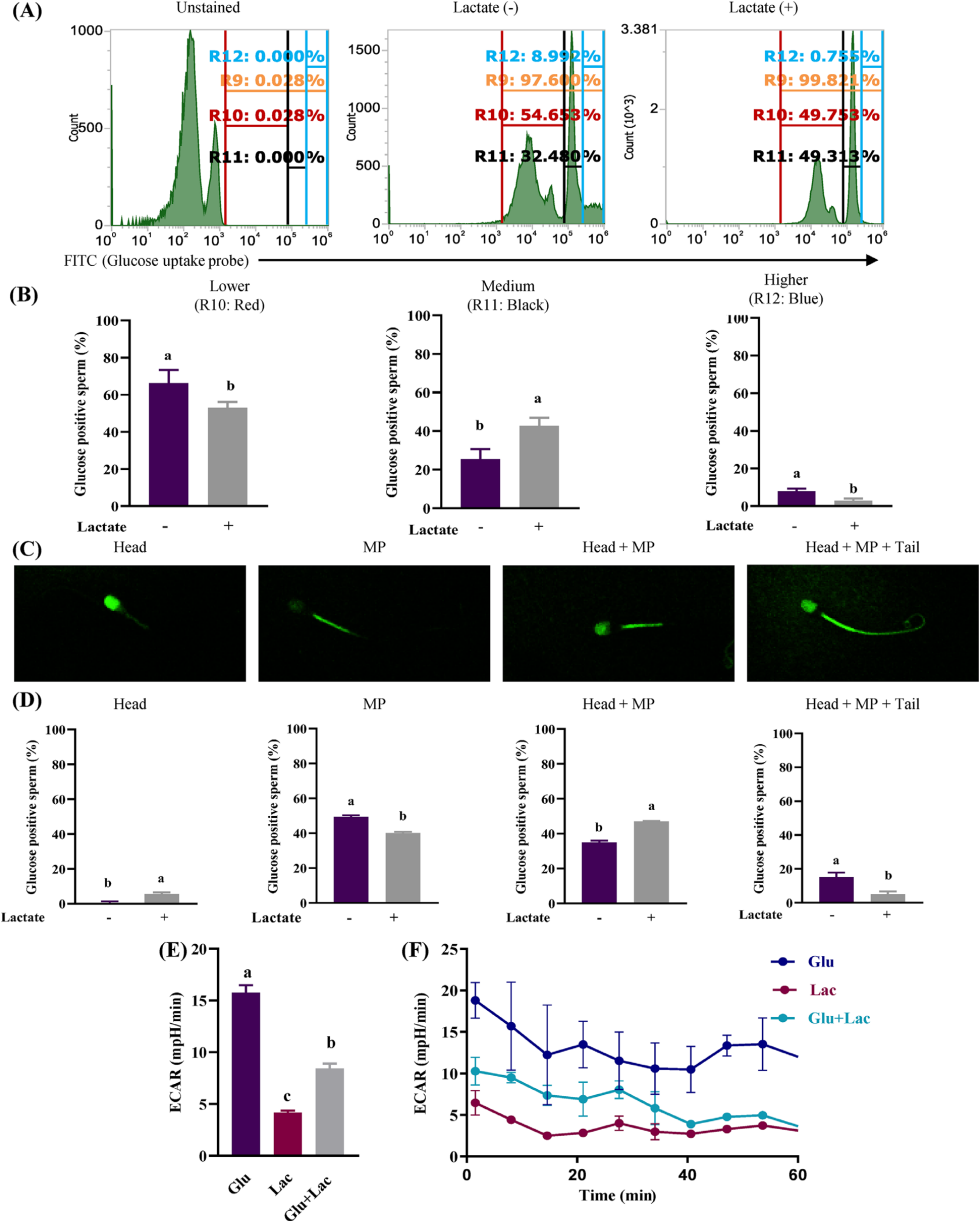
Effects of lactate on glucose uptake and glycolysis. Cellular glucose uptake was evaluated in the absence and presence of lactate with a glucose uptake assay kit by flow cytometry and confocal microscopy. FITC‐positive spermatozoa indicate uptake of fluorescent‐labeled glucose. There are differences in fluorescence intensity among the sperm population, and it was categorized into three groups (i.e., low, medium, and high) based on the intensity (A, B). Four positive staining patterns (head, midpiece; MP, head + MP, head + MP + tail) of glucose‐positive spermatozoa were identified by the confocal microscope (C). Percentage of FITC‐positive spermatozoa (D). Cellular glycolysis was measured by flux analysis (E, F). Data are presented as mean ± SD from three replicates. Different lowercase subscripts indicate significant differences (*p* < 0.05). a.u., arbitrary units; ECAR, extracellular acidification rate; Glu, glucose; Lac, lactate; MP, midpiece.

To investigate whether the changes in sperm kinematics were linked to glycolysis, a fluorescence‐based glucose uptake assay was used to determine sperm efficiency in incorporating glucose in lactate‐free and with‐lactate conditions. Flow cytometry data showed that almost all spermatozoa took up glucose, but it was found that subgroups appeared based on differences in fluorescence intensity (Figure 5A; R9 gate). When the positive gate was analyzed into three groups (Lower, Medium, and Higher: Figure 5A; R10‐12 gate), the Higher group of spermatozoa was reduced when lactate was added. Conversely, more spermatozoa from the Lower group were observed in the absence of lactate (Figure 5B). Spermatozoa observed under the confocal microscope were classified into one of four patterns based on fluorescent localization: head only, midpiece only, head and midpiece, or head, midpiece, and tail (Figure 5C). In the presence of lactate, glucose uptake in the midpiece or tail was significantly suppressed, while the percentage of spermatozoa that took up glucose in the head increased (Figure 5D). To investigate how these changes in glucose uptake patterns affected glycolysis, ECAR was measured using the flux analyzer. There was a significant reduction in cellular ECAR when spermatozoa were cultured with both glucose and lactate compared with glucose alone (*p* < 0.05; Figure 5E,F).

### 3.5 Glucose‐derived acrosome reaction is suppressed by lactate

**FIGURE 6:**
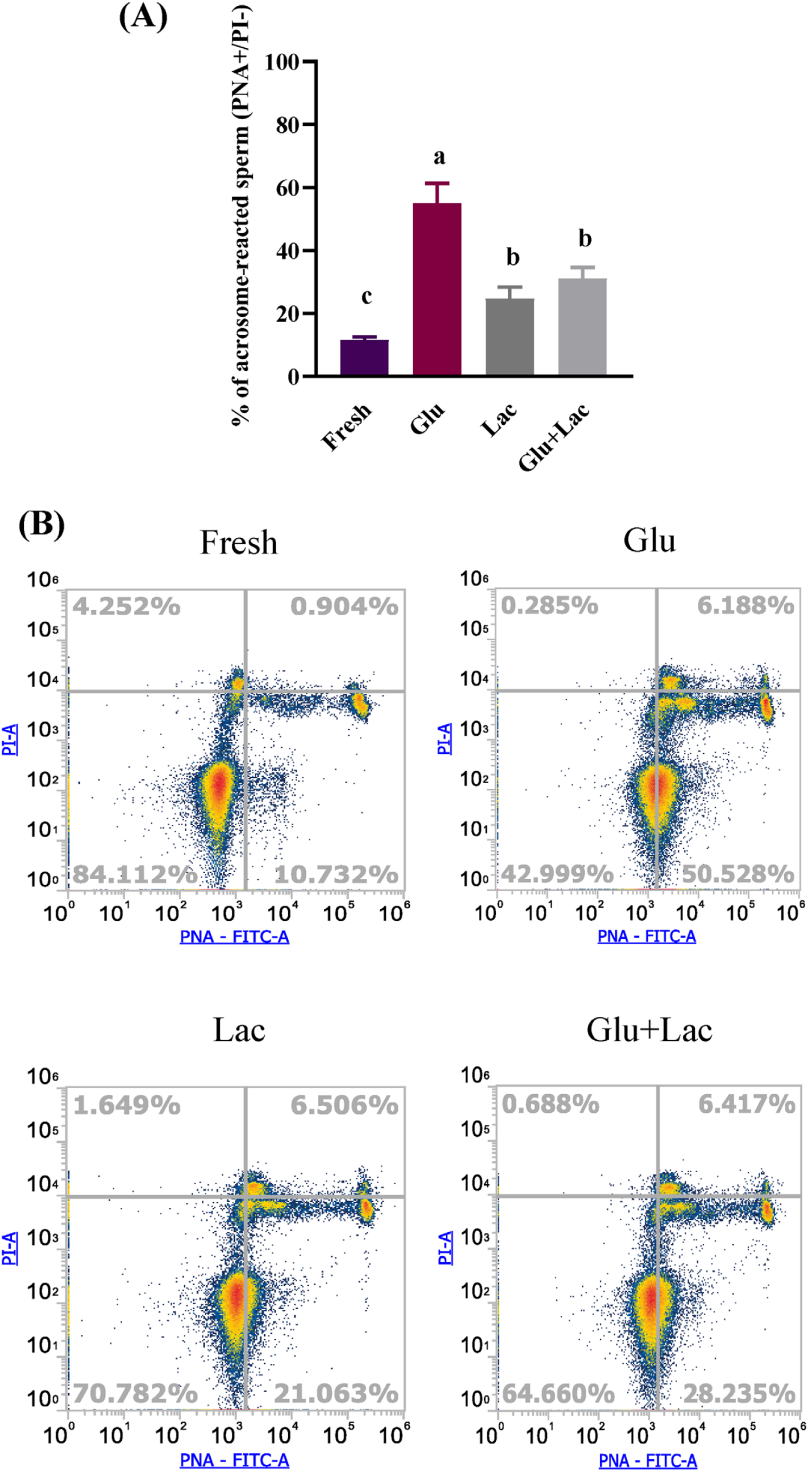
Glucose‐derived acrosome reaction is suppressed by lactate. Acrosome reactivity of fresh spermatozoa and glucose, lactate, and glucose + lactate‐treated spermatozoa was evaluated by PNA/PI. The percentage of PNA+/PI− was considered as acrosome‐reacted spermatozoa (lower right). Glucose treatment increases the acrosome‐reacted spermatozoa (%), whereas glucose + lactate treatment reduces the acrosome‐reacted spermatozoa (%) (A). Representative flowcytometry image of live/dead and acrosome intact/reacted spermatozoa of different treatment group (B). Data are presented as mean ± SD from three replicates. Different lowercase subscripts indicate significant differences when *p* < 0.05. Glu, glucose; Lac, lactate; PI, propidium iodide; PNA, peanut agglutinin.

Lactate reduced glycolysis and thus shifted the sperm energy metabolism from glycolysis to OXPHOS. To investigate the possibility that reduced glycolysis might have effects on sperm acrosome reaction, we evaluated the acrosome reactivity of spermatozoa in the presence of different substrates. Spermatozoa cultured solely with glucose showed a higher percentage of reacted acrosomes compared with those incubated with lactate (*p* < 0.05). The combination of glucose and lactate resulted in a significant reduction in acrosome reactivity, suggesting that lactate impairs glucose‐induced acrosome reactions (Figure 6A–E).

## 4 DISCUSSION

**FIGURE 7:**
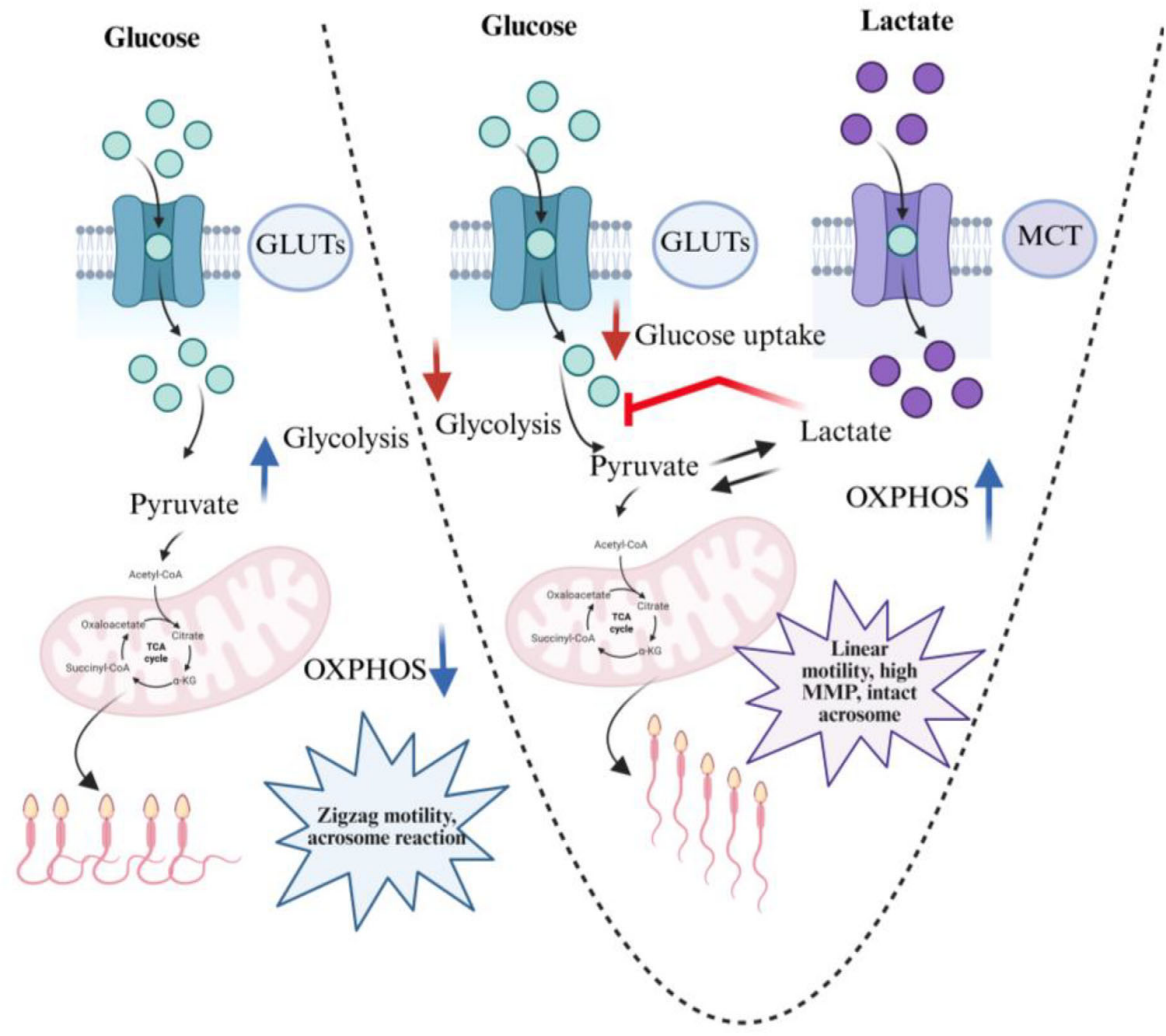
Schematic illustration of the dual role of lactate in bovine sperm metabolism and functionality. Spermatozoa take up glucose and metabolize it through glycolysis. Adenosine triphosphate (ATP) generated through glycolysis energizes the sperm flagellum, resulting in a zigzag motility marked by higher VCL, ALH, and higher percentages of acrosome‐reacted spermatozoa. When lactate is added in the presence of glucose, it suppresses glycolysis and enhances oxidative phosphorylation (OXPHOS). Reduced glycolysis and enhanced OXPHOS result in higher linear motility, elevated mitochondrial membrane potential (MMP), and higher acrosomal integrity. Thus, besides its conventional role of metabolic substrates, lactate regulates glucose metabolism and maintains sperm functionality.

Mammalian spermatozoa ejaculated in the female reproductive tract along with seminal plasma to fertilize the oocyte in vivo.^2^ Immediately after ejaculation, spermatozoa must travel through the female tract to meet the oocyte ovulated at the far end of the oviduct for successful fertilization.^20^ The spermatozoa shows two different motility patterns during this journey, linear motility in the uterus to maintain sperm transportation and zigzag motility in the oviduct, necessary for fertilization.^21^ It has been well established across the species that spermatozoa maintain their motility and functionality with a well‐balanced metabolic mechanism that produces ATP through glycolysis in the cytoplasm or OXPHOS in the mitochondria or a combination of both.^8^, ^22^ In bull spermatozoa, both glycolytic and non‐glycolytic energy substrates can be used efficiently to generate sufficient ATP required for motility and functionality.^22^ However, there is an interesting interplay between the role of glucose and lactate in different mammalian cell types and recent studies show that Tricarboxylic Acid (TCA) cycles carbon sources are fueled by lactate, not glucose.^23^, ^24^ In bull spermatozoa, lactate can maintain sperm motility similar or even better than glucose.^25^ Our research revealed an interesting insight into the interplay between glucose and lactate in modulating bull sperm energy metabolism and thus regulating sperm function (Figure 7).

Glucose is utilized through glycolysis and maintains hyperactivated motility, whereas lactate oxidizes through OXPHOS and results in linear motility.^20^, ^26^ Reduced glucose concentration in the medium increased sperm mitochondrial activity that shifts the motility pattern from zigzag to linear, indicating that spermatozoa can sense the changes in substrate and adjust the metabolic pathways that modulate their motility pattern while keeping their total motility unchanged.^22^ This type of metabolic plasticity allows the spermatozoa to survive and maintain its functionality in diverse metabolic conditions in different anatomic parts of the female tract. When we treated the spermatozoa with the combination of glucose and lactate, it did not show any synergistic effects on sperm motility and kinematics; instead, sperm characteristics were as similar to the lactate alone condition. Later, we identified that, in the presence of lactate, glucose incorporation in the midpiece and tail regions is suppressed, and glycolysis is reduced. Spermatozoa responds to this suppression by shifting the metabolic pathways from glycolysis to OXPHOS. The metabolic transition is evidenced by the alteration from zigzag to linear motility patterns. To understand the possible mechanism of this suppressive role of lactate we need to understand how lactate is utilized by the spermatozoa. Extracellular lactate is uptaken by spermatozoa and converted to pyruvate, which enters the TCA cycle to produce ATP.^27^ The entire process enhances the downstream pathways of the TCA cycle and sends negative feedback to suppress the upstream pathways of glycolysis. A recent study from our laboratory shows that itaconate an intermediate metabolite of the TCA cycle inhibits glycolysis and enhances OXPHOS and pentose phosphate pathway which generate NADPH and result in linear motility in boar spermatozoa.^28^

Lactate might also inhibit glycolysis by modifying the NADH/NAD⁺ ratio and decreasing the activity of essential glycolytic enzymes, including glyceraldehyde‐3‐phosphate dehydrogenase (GAPDH).^24^, ^29^ These types of metabolic alterations have been observed in somatic cells, where lactate inhibits glycolysis and promotes mitochondrial respiration via the lactate shuttle mechanism.^30^, ^31^, ^32^ Moreover, the inhibition of glucose uptake by lactate may result from competitive interference with glucose transporters (GLUTs) or metabolic changes mediated by monocarboxylate transporters (MCTs).^33^ In boar spermatozoa, lactate is favored over other glycolytic substrates, and its presence suppresses the metabolism of glucose and fructose, indicating a feedback mechanism that reduces glycolysis when lactate levels are elevated.^34^, ^35^ Lactate and pyruvate are the most preferred metabolic substrates over glucose in equine spermatozoa.^36^ The results of our study not only confirmed the importance of lactate in ATP production in spermatozoa but also showed that lactate inhibited glucose uptake and glycolysis in spermatozoa as somatic cells.^37^ Additionally, it is clarified that the changes in the glucose utilization mechanism by lactate affected the motility pattern of spermatozoa.

The biological implications of these metabolic interplay might be useful for understanding sperm functions. Our findings indicate that lactate affects glucose‐induced acrosome reaction. The decrease of acrosome‐reacted spermatozoa in lactate‐rich conditions corresponds with previous research demonstrating that ATP generated from glycolysis is essential for hyperactivation and acrosomal exocytosis.^22^, ^38^ Glucose is an essential substrate for generating ATP and other signaling molecules required for the acrosome reaction,^39^ whereas lactate, pyruvate, and hydroxybutyrate can maintain comparatively lower levels of tyrosine phosphorylation and hyperactivation than glucose.^40^ However, if the spermatozoa becomes acrosome reacted in the uterus or oviduct before meeting the oocytes it may impair sperm function.^41^ Early acrosome‐reacted spermatozoa binds with the uterine and oviductal surface during transportation and reduces the chances of fertilization.^42^ Lactate might play a regulatory role in maintaining acrosome intactness by inhibiting glycolysis until the spermatozoa reaches the site of fertilization to meet the oocyte. However, spermatozoa might need functional modifications to attain the fertilization status after reaching the site of fertilization.

## 5 CONCLUSION

In conclusion, this study reveals the dual role of lactate as an energy substrate and a regulator of glucose metabolism in bull spermatozoa. Lactate might be considered as an essential component of sperm handling, storage, and thawing medium to maintain the viability and functionality of spermatozoa. These novel findings might improve our understanding of the study of sperm physiology to create opportunities for advancements in sperm preservation and assisted reproductive technology (ART).

## AUTHOR CONTRIBUTIONS

**Md Faizul Hossain Miraz**: Writing the original draft, methodology, data curation, analysis, and visualization; **Takahiro Yamanaka**: Methodology, visualization, review, and editing; **Shahrina Akter**: Review and editing; **Masanori Koyago**: Methodology and data curation; **Masayuki Shimada**: Conceptualization, review and editing, investigation, and funding acquisition.

## CONFLICT OF INTEREST STATEMENT

Masayuki Shimada holds the position of board member of Hiroshima Cryopreservation Service. Masayuki Shimada acts as a consultant or advisor in Rohto Pharmaceutical Co. Ltd. Masayuki Shimada has connections with the Bill & Melinda Gates Foundation and received funding grants. Other authors declare no conflicts of interest.

## Supporting information



Supporting Information

## Data Availability

All data relevant to this study are included in the manuscript or Supporting Information file.
