# Supplementary material for: Lactate inhibits glucose‐induced zigzag motility and enhances linear motility in bull spermatozoa by suppressing glycolysis
Source: Andrology. 2025 Aug 15;14(3):933–45. doi: 10.1111/andr.70113 (PMC12917573; doi:10.1111/andr.70113)
Supplement: Supplementary file 1 — Supporting Information [file ANDR-14-933-s001.docx]

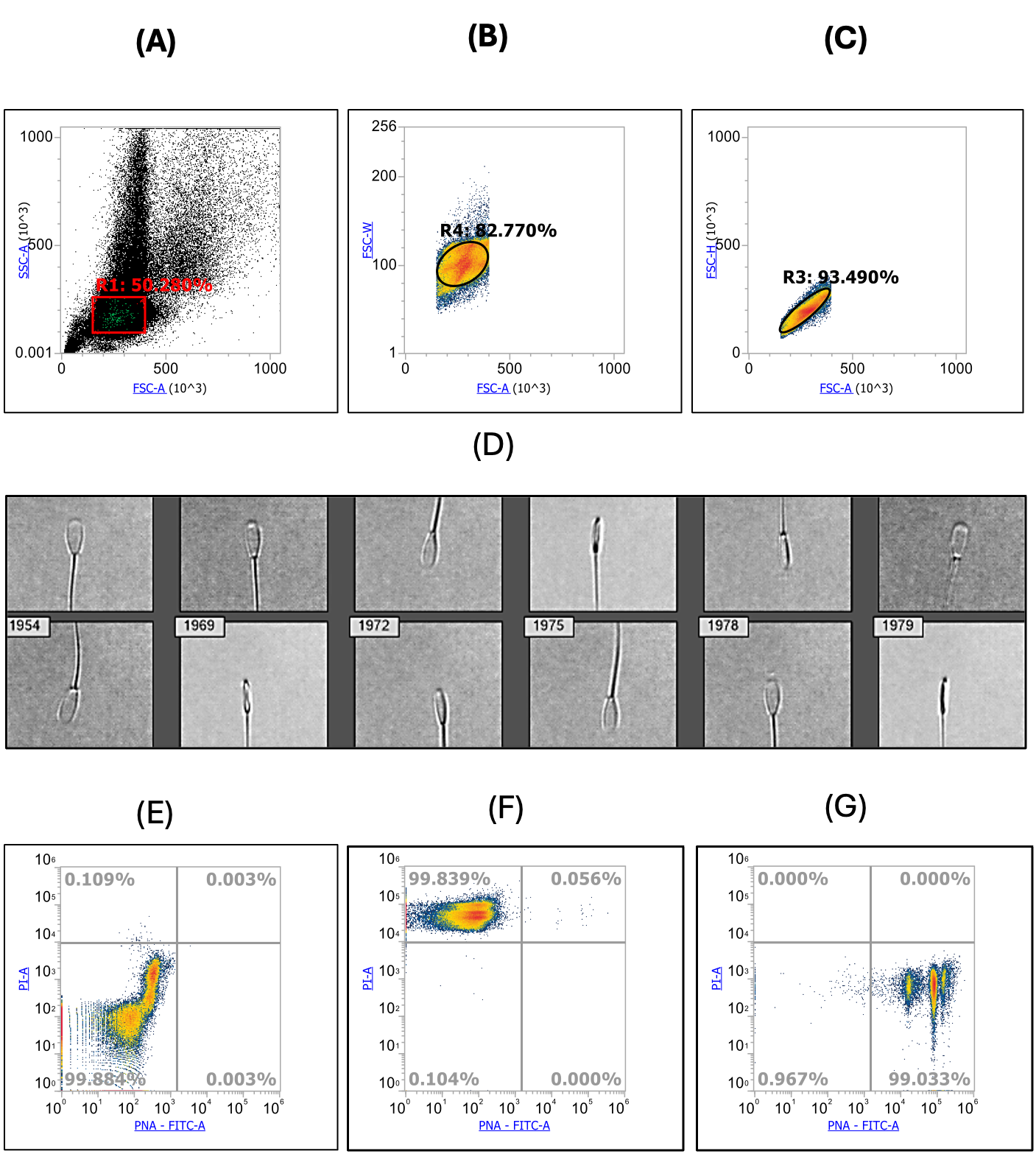


**Supplementary Figure 1. Flow cytometry gating strategy**

Sperm cells of equal size and complexity were identified by forward scatter (FSC-A) and side scatter (SSC-A) dot plots (R1) (A) , Subsequently, FSC-A vs FSC-W (R4) and FSC-A versus FSC-H (R3) dot plots enhance the selection by aggregating cells of similar size inside designated regions (R4, R3) (B-C). Data analysis was conducted from R3 (C). Representative image of single sperm selection by gating (D). Gating strategy for sperm viability (PI) and acrosome integrity (PNA) analysis (E-F) . Quadrant gates were set using controls to distinguish four populations: live acrosome-intact (PI⁻/PNA⁻, lower-left), dead acrosome-intact (PI⁺/PNA⁻, upper-left), dead acrosome-reacted (PI⁺/PNA⁺, upper-right), and live acrosome-reacted (PI⁻/PNA⁺, lower-right). An unstained sample defines the PI⁻/PNA⁻ population (E). A damaged sperm membrane (0.3% Triton treated) sample helps position the horizontal gate for dead (PI⁺) cells (F). A permeabilized positive control (0.3% Triton treated) generates a distinct PNA⁺ population. Combination of unstained, PI⁺ and PNA⁺ were used to precisely set the final gate boundaries.

**Supplementary Table 1.** Modified HTF medium composition (mM)

| **Component** | **No Energy** | **Glu** | **Lac** | **Glu + Lac** |
| --- | --- | --- | --- | --- |
| NaCl | 93.1 | 93.1 | 93.1 | 93.1 |
| KCl | 4.7 | 4.7 | 4.7 | 4.7 |
| MgSO₄·7H₂O | 0.2 | 0.2 | 0.2 | 0.2 |
| KH₂PO₄ | 0.37 | 0.37 | 0.37 | 0.37 |
| Glucose | 0 | 2.8 | 0 | 2.8 |
| Na-Lactate | 0 | 0 | 23.6 | 23.6 |
| CaCl₂·2H₂O | 5.1 | 5.1 | 5.1 | 5.1 |
| NaHCO₃ | 25.0 | 25.0 | 25.0 | 25.0 |
| Na-Pyruvate | 0 | 0 | 0 | 0 |
| Streptomycin | 0.086 | 0.086 | 0.086 | 0.086 |
| Penicillin G | 0.19 | 0.19 | 0.19 | 0.19 |
| BSA | 0 | 0 | 0 | 0 |
| HEPES | 25.0 | 25.0 | 25.0 | 25.0 |
